# Antibody Response against SARS-CoV-2 and Seasonal Coronaviruses in Nonhospitalized COVID-19 Patients

**DOI:** 10.1128/mSphere.01145-20

**Published:** 2021-02-24

**Authors:** Natalia Ruetalo, Ramona Businger, Karina Althaus, Simon Fink, Felix Ruoff, Michaela Pogoda, Angelika Iftner, Tina Ganzenmüller, Klaus Hamprecht, Bertram Flehmig, Tamam Bakchoul, Markus F. Templin, Michael Schindler

**Affiliations:** a Institute for Medical Virology and Epidemiology of Viral Diseases, University Hospital Tübingen, Tübingen, Germany; b Institute for Transfusion Medicine, University Hospital Tübingen, Tübingen, Germany; c NMI, Reutlingen, Germany; d Institute of Medical Genetics and Applied Genomics, University of Tübingen, Tübingen, Germany; e NGS Competence Center Tübingen, Tübingen, Germany; f Mediagnost GmbH, Reutlingen, Germany; National Institute of Allergy and Infectious Diseases

**Keywords:** COVID-19, SARS-CoV-2, antibody-dependent enhancement (ADE), neutralizing antibodies, seasonal coronaviruses

## Abstract

The majority of infections with SARS-CoV-2 are asymptomatic or mild without the necessity of hospitalization. It is of importance to reveal if these patients develop an antibody response against SARS-CoV-2 and to define which antibodies confer virus neutralization. We conducted a comprehensive serological survey of 49 patients with a mild course of disease and quantified neutralizing antibody responses against a clinical SARS-CoV-2 isolate employing human cells as targets. Four patients (8%), even though symptomatic, did not develop antibodies against SARS-CoV-2, and two other patients (4%) were positive in only one of the six serological assays employed. For the remaining 88%, antibody response against the S protein correlated with serum neutralization whereas antibodies against the nucleocapsid were poor predictors of virus neutralization. None of the sera enhanced infection of human cells with SARS-CoV-2 at any dilution, arguing against antibody-dependent enhancement of infection in our system. Regarding neutralization, only six patients (12%) could be classified as high neutralizers. Furthermore, sera from several individuals with fairly high antibody levels had only poor neutralizing activity. In addition, employing a novel serological Western blot system to characterize antibody responses against seasonal coronaviruses, we found that antibodies against the seasonal coronavirus 229E might contribute to SARS-CoV-2 neutralization. Altogether, we show that there is a wide breadth of antibody responses against SARS-CoV-2 in patients that differentially correlate with virus neutralization. This highlights the difficulty to define reliable surrogate markers for immunity against SARS-CoV-2.

**IMPORTANCE** There is strong interest in the nature of the neutralizing antibody response against SARS-CoV-2 in infected individuals. For vaccine development, it is especially important which antibodies confer protection against SARS-CoV-2, if there is a phenomenon called antibody-dependent enhancement (ADE) of infection, and if there is cross-protection by antibodies directed against seasonal coronaviruses. We addressed these questions and found in accordance with other studies that neutralization is mediated mainly by antibodies directed against the spike protein of SARS-CoV-2 in general and the receptor binding site in particular. In our test system, utilizing human cells for infection experiments, we did not detect ADE. However, using a novel diagnostic test we found that antibodies against the coronavirus 229E might be involved in cross-protection to SARS-CoV-2.

## INTRODUCTION

The most recent emerging virus outbreak happened in China in December 2019, caused by SARS (severe acute respiratory syndrome) coronavirus 2 (SARS-CoV-2) (1), leading to a pandemic, as defined by the WHO in March 2020 (2, 3). Infections with SARS-CoV-2 can cause the so-called disease COVID-19 (coronavirus disease 2019). Fifty percent of all COVID-19 cases range from asymptomatic to mild. Thirty percent show moderate to pronounced symptoms. Five to 20% of patients are hospitalized due to critical course of infection with severe lung complications, and on average, ∼5% die, even though there is high variation dependent on the country (4). Recent data from a multicentric cohort of 10,021 hospitalized COVID-19 patients showed an in-hospital mortality of 73% in mechanically ventilated patients requiring dialysis and of 53% of invasively ventilated patients (5). One critical determinant of illness is age, as mortality is highest in the elderly population (1, 2, 4, 5). SARS-CoV-2 is currently spreading in an immune-naive population, and a vaccine was not yet available at the time of the writing of the manuscript, but there were numerous candidates in the advanced development pipeline (6). For an updated online resource, refer to https://biorender.com/covid-vaccine-tracker.

The pandemic is not only devastating in terms of the direct harm to human health inflicted by the virus infection, but the continuous quarantine and lockdown measures have enormous negative impact on the socioeconomic life of billions of individuals (7). Furthermore, numerous studies assessing the prevalence of SARS-CoV-2-specific antibodies in the population have been initiated (8–11).

Regardless of the prevalence of antibodies against SARS-CoV-2, one still poorly defined determinant is what type of antibodies neutralize SARS-CoV-2 and hence potentially confer protective immunity against the infection, even though very recent data using Vero cells and pseudovirus systems suggest that IgGs against the receptor binding domain (RBD) play a role (12, 13). Besides, antibodies that bind to SARS-CoV-2 but do not result in neutralization might enhance infection, a phenomenon called antibody-dependent enhancement (ADE) (14, 15), which has not been fully investigated. Finally, the role of cross-protecting antibodies from seasonal coronaviruses is also discussed but not yet experimentally assessed (14).

To shed further light on the determinants of human serum in neutralizing SARS-CoV-2, we performed a comprehensive serological analysis of 49 individuals who were nonhospitalized and ranged from an asymptomatic to a mild course of disease. We employed several assays measuring SARS-CoV-2-specific IgGs against the S protein, the S protein RBD (S-RBD), and the nucleocapsid. Furthermore, we assessed S-RBD-specific IgM and IgA and used a novel high-throughput Western blot system to detect IgGs against SARS-CoV-2 and seasonal coronaviruses. Finally, all serological parameters were associated with the ability of the 49 sera to neutralize the infection on human cells with a clinical SARS-CoV-2 isolate.

## RESULTS

### The majority of patients develop SARS-CoV-2-specific antibodies.

For our serological survey, we recruited individuals coming to the Department of Transfusion Medicine to donate blood for plasma therapy. All 49 patients included in this study were nonhospitalized with asymptomatic to mild courses of disease, including cough (69%), fever (59%), limb pain and headache (35%), diarrhea (10%), and loss of taste (10%) (see Table S1 in the supplemental material). The age ranged from 19 to 66 years (median, 40 years), and gender was balanced (24 male, 25 female). The time from positive SARS-CoV-2 test to blood sampling was 14 to 64 days (median, 45 days).

10.1128/mSphere.01145-20.1 TABLE S1 Primary data of serological tests and patient characteristics. Download Table S1, XLSX file, 0.01 MB. Copyright © 2021 Ruetalo et al. 2021 Ruetalo et al. https://creativecommons.org/licenses/by/4.0/ This content is distributed under the terms of the Creative Commons Attribution 4.0 International license.

We employed several serological assays to detect antibodies against SARS-CoV-2 (Table 1 and Table S1). IgG enzyme-linked immunosorbent assays (ELISAs) against the S1 protein (Euroimmun) and S-protein RBD (Mediagnost) and IgA and IgM against S-RBD (Mediagnost) as well as an electrochemiluminescence immunoassay (ECLIA) detecting IgG against the viral nucleocapsid (NC; Roche). Furthermore, we applied a throughput Western blot system (DigiWest) allowing detection of SARS-CoV-2 and seasonal coronavirus antibodies (16).

**TABLE 1:**
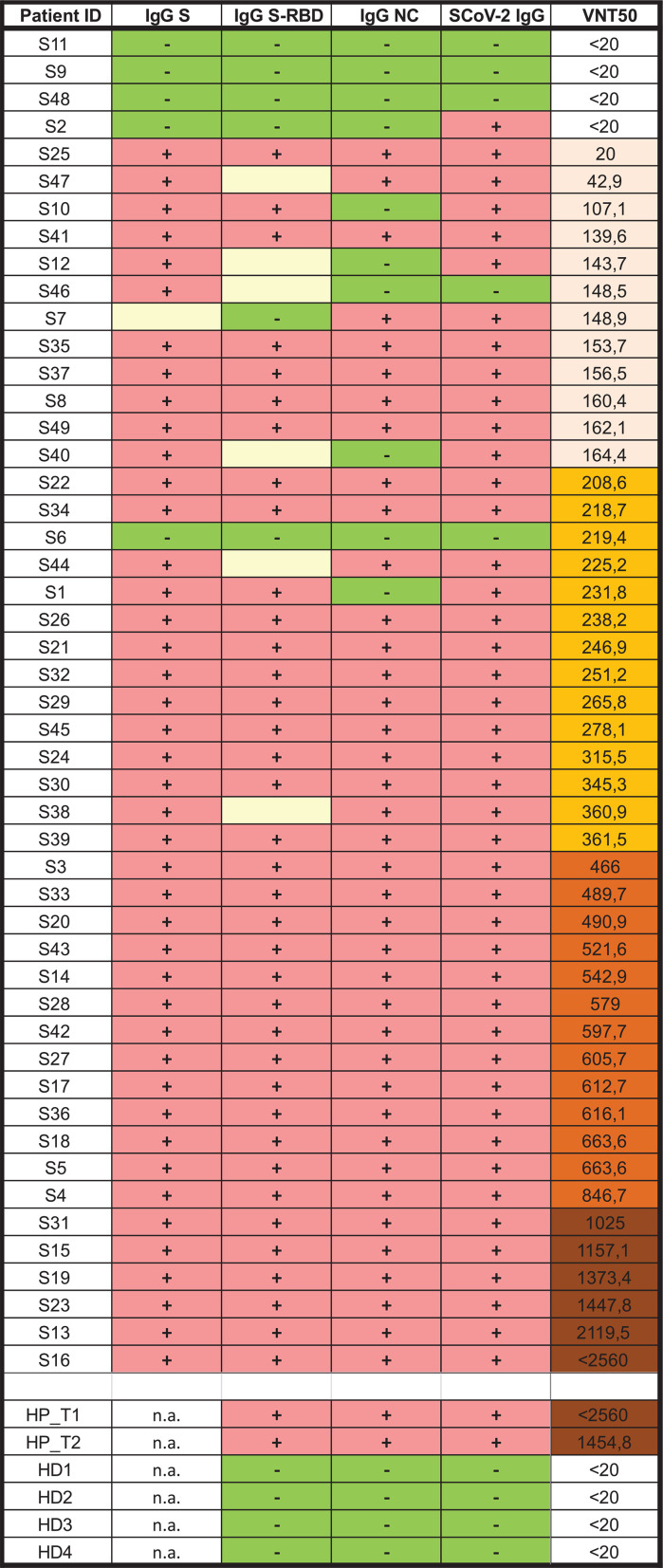
Serological parameters of sera and VNT_50_ values^*a*^

a Sera of the 49 patients are ordered by their VNT_50_ and color coded. For the serological tests indicated, there is a qualitative indicator for a value above the threshold (+), below the threshold (−), or borderline (0) for the respective assay. At the bottom of the table, the control sera are shown: two consecutive sera of a hospitalized patient (HP_T1/T2) and four sera of healthy donors (HD1 to 4). All the individual values and the specific patient characteristics are listed in Table S1.

Four of 49 (8%) sera (S6, S9, S11, and S48) were negative in all serological assays employed to detect SARS-CoV-2-specific antibodies, even though they were positively tested by PCR and patients were symptomatic, showing two or more symptoms. In addition, two more sera (S2 and S46) were positive in only one of the four assay systems to detect IgGs against S or NC, the reason why we consider these sera also negative. Of note, three patients who did not provide a PCR-confirmed diagnosis of SARS-CoV-2 (S10/S22/S44) were shown to be infected, judging by the presence of specific antibodies against SARS-CoV-2 in all of them as well as neutralization ability (virus-neutralizing titer 50 [VNT_50_],107 to 255) (Table S1). Apart from the robust IgG response against both the S protein (90%) and NC (80%), development of S-specific IgM and IgA was less prominent, with 35 and 30% of positive sera, respectively (Table S1).

In sum, even though 12% (6/49) of patients did not develop detectable antibodies against SARS-CoV-2, the vast majority (88%, 43/49) of individuals mounted a robust SARS-CoV-2-specific antibody response.

### Few patients develop high virus-neutralizing titers (VNTs) after SARS-CoV-2 infection.

**FIG 1:**
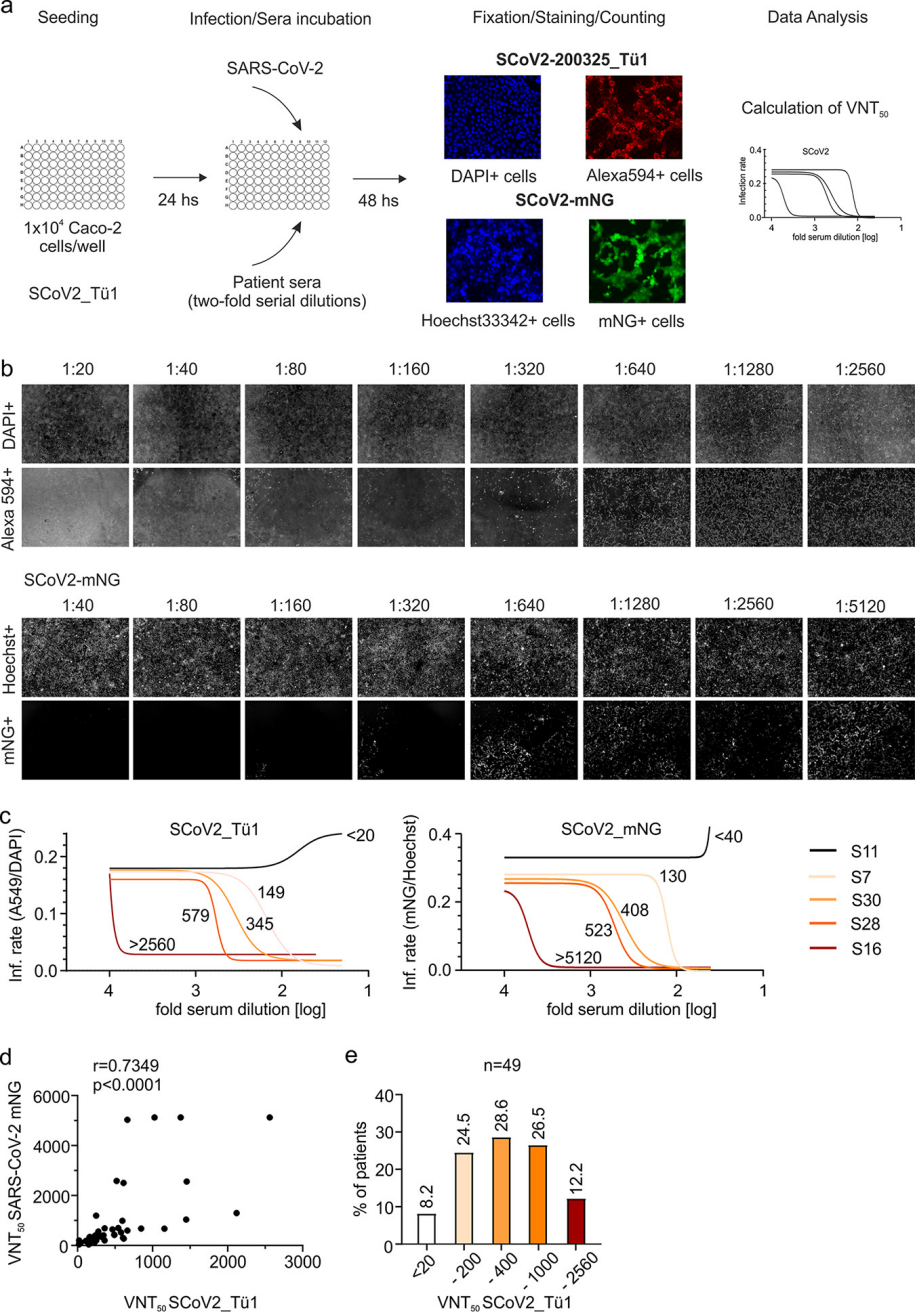
Neutralization of SARS-CoV-2 by sera of COVID-19 convalescent patients. (a) Experimental layout of the two neutralization assays employed using the clinical isolate (SARS-CoV-2_Tü1) and the green fluorescent virus (SARS-CoV-2_mNG). (b) Primary data showing results of both neutralization assays using one patient serum as an example (S28). In the upper row of each set, the total amount of cells for each well of the 2-fold serial dilution of sera is shown, as DAPI^+^/Hoechst^+^, respectively. In the lower row of each set, infected cells are visualized, indicated as Alexa 594^+^/mNG^+^ cells, respectively. (c) Neutralization curves of five representative sera measured by both assays. The graphs show the nonlinear regression fitting calculated for five patients who displayed different neutralization capacity: no, poor, low, medium, and high neutralization. The VNT_50_ for each patient is shown next to each curve. (d) Correlation analysis of VNT_50_ measured by both assays (*n* = 49). Correlation is calculated as Pearson’s *r*. (e) Percentage of patients classified according to the VNT_50_ using SARS-CoV-2_Tü1. The titers used to classify the sera are shown below the columns: <20, 20 to 200, 201 to 400, 401 to 1,000, and 1,000 to 2,560. Above the columns is shown the percentage of sera that correspond to each category.

The majority of SARS-CoV-2-infected individuals seroconvert within 14 days (27); however, it is less clear how potently sera from these patients neutralize SARS-CoV-2 (17). To test for virus neutralization, we established two procedures using human Caco-2 cells as targets (Fig. 1a). First, we infected cells with a SARS-CoV-2 strain isolated from a throat swab of a patient showing a high viral load as determined by reverse transcription-quantitative PCR (qRT-PCR). This strain was designated SARS-CoV-2 200325_Tü1. Cells were coincubated with patient sera and virus in serial 2-fold dilutions from 1:20 up to 1:2,560. At 48 h postinfection (hpi), cells were fixed with 80% acetone and immunofluorescence stained against SARS-CoV-2 antigens with a highly potent patient serum we retrieved from a hospitalized convalescent donor. Cells were counterstained with 4′,6-diamidino-2-phenylindole (DAPI), and infection rates were quantified via automated fluorescence microscopy. For the second approach, we employed the mNeonGreen (mNG)-expressing infectious SARS-CoV-2 clone (12). Cells were treated and infected exactly as explained for the SARS-CoV-2 Tü1 strain, but using slightly adjusted dilutions of sera; 1:40 to 1:5,120. At 48 hpi, cells were fixed with 2% paraformaldehyde (PFA) containing Hoechst 33342 as nuclear stain (Fig. 1b, representative serum examples of both procedures). Infection rates of the corresponding serum dilutions were used to plot sigmoidal inhibition curves and calculate the virus-neutralizing titer 50 (VNT_50_), which is the serum dilution inhibiting the half-maximal infection (Fig. 1c). The VNT_50_ values of the sera obtained with the primary patient isolate strongly correlated with the titers calculated when using the mNeonGreen-expressing infectious clone (*r* = 0.7349; Fig. 1d). We obtained only slight discrepancies for highly potent sera that seemed to neutralize SARS-CoV-2-mNG more efficiently than SARS-CoV-2 Tü1 at high dilutions. In both assays, sera from four healthy donors and two consecutive sera from a hospitalized convalescent patient either were completely negative for VNT as well as all serological assays or showed robust neutralization and SARS-CoV-2-specific antibodies (Table 1).

Patient sera were classified according to their neutralizing capacity (Fig. 1e), revealing that only 12% (6/49) were highly potent neutralizers. Eight percent (4/49) of sera did not neutralize SARS-CoV-2 at all *in vitro*, and 24% (12/49) were poor neutralizers even though showing robust signals in the different serological assays employed. Of note, using human Caco-2 cells that express human Fc receptors (18), we should be able to observe ADE. However, none of the sera enhanced infection of SARS-CoV-2 at any dilution, arguing against ADE, at least in our system (see examples in Fig. 1b and c). Hence, there is a large diversity in the ability of patient sera to neutralize SARS-CoV-2 which is not always associated with the amount of SARS-CoV-2-specific antibodies.

### VNT_50_ is not associated with specific patient characteristics, apart from gender.

**FIG 2:**
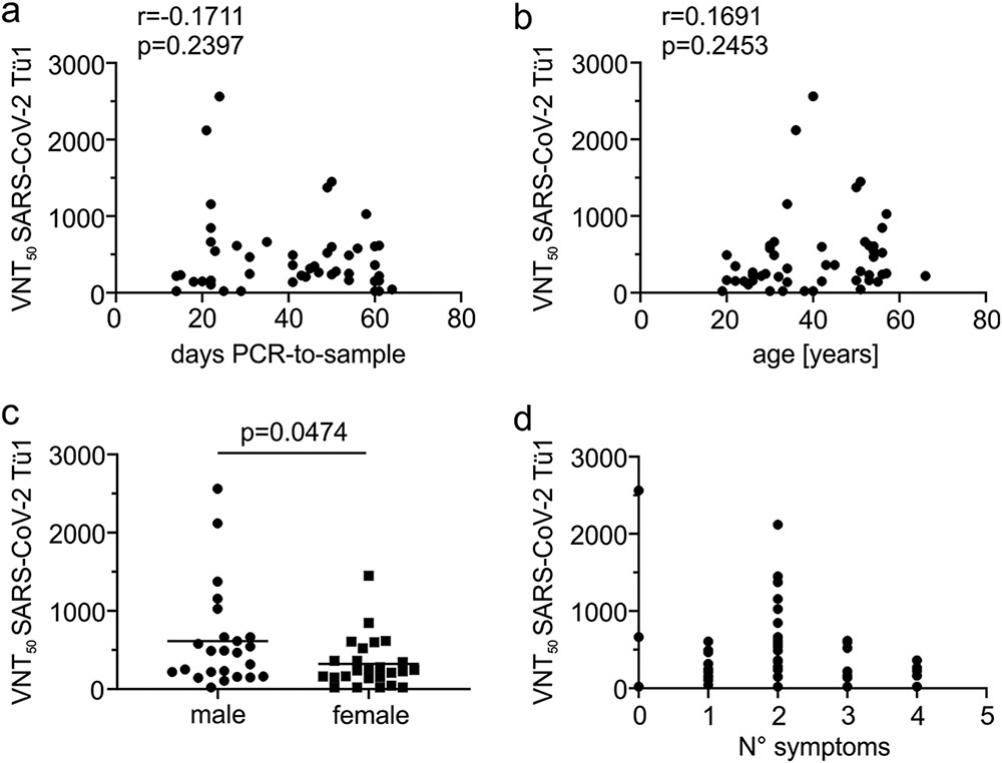
Association of patient characteristics with serum VNT_50_. The VNT_50_ of each patient serum was associated with the individual date of the positive SARS-CoV-2 qRT-PCR diagnostic test to blood sampling (a), the age of the patient (b), the gender (c), and the number of symptoms reported (d). Statistical analyses were done with an unpaired two-tailed Student *t* test; correlations are calculated as Pearson’s *r*. See detailed patient characteristics in Table S1.

We next assessed potential associations of patient characteristics with serum neutralization. Neither the sampling date 14 to 64 days (Fig. 2a) nor patient age (Fig. 2b) correlated with serum neutralization. This indicates that seroconversion, as reported, is achieved within 14 days in all patients and VNTs might not drop for up to 64 days. On average, titers were higher in males than females (Fig. 2c). In detail, 3/4 individuals who did not neutralize at all were female but 5/6 highly potent neutralizers were male (Table S1). Furthermore, the average VNT_50_ of men is double that of women (613 ± 130 versus 322 ± 64 [standard error of the mean {SEM}]; compare Fig. 2c). Of note, there was no association of VNT_50_ with the severity of disease (Fig. 2d), that is, amount of symptoms.

### SARS-CoV-2-specific IgG against the S-protein RBD indicates serum neutralization.

**FIG 3:**
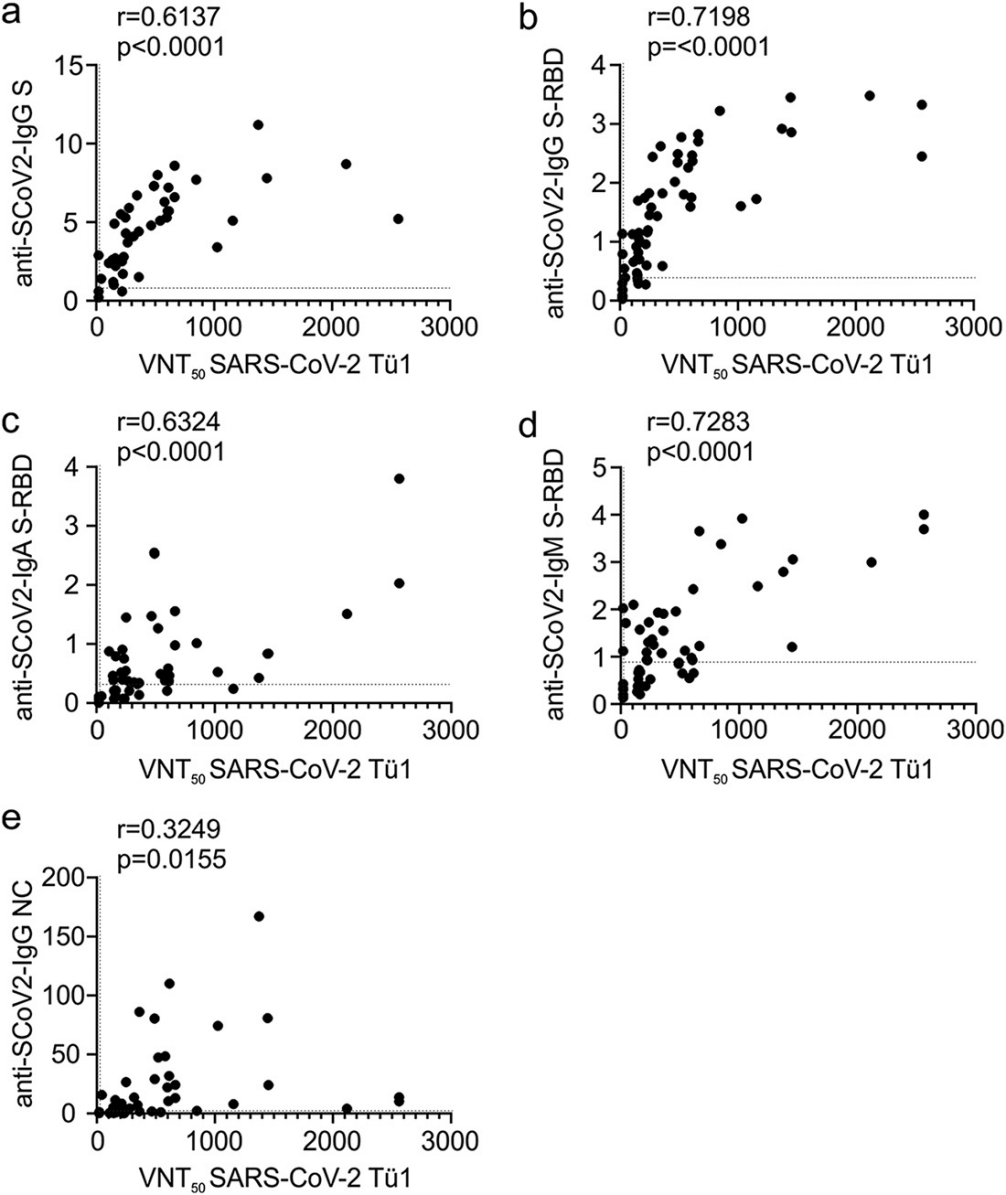
Correlation of serological parameters with serum VNT_50_. The VNT_50_ of each patient serum was correlated with the value of SARS-CoV-2 S-specific IgGs measured by the Euroimmun ELISA (a), the relative quantitative value of SARS-CoV-2 S-RBD-specific IgGs measured by the Mediagnost ELISA (b), the relative quantitative value of SARS-CoV-2 S-RBD-specific IgAs measured by the Mediagnost ELISA (c), the relative quantitative value of SARS-CoV-2 S-RBD-specific IgMs measured by the Mediagnost ELISA (d), and the relative quantitative value of SARS-CoV-2 NC-specific IgGs measured by the Roche ECLIA (e). Dotted lines indicate the assay thresholds. Correlations are calculated as Pearson’s *r*.

Next, we set out to define serological correlates of virus neutralization *in vitro*. Overall, the neutralizing capacity of the sera correlated with the abundance of SARS-CoV-2-specific IgG against the S protein (*r* = 0.6137 [Fig. 3a]), with a slightly better *r* value when the IgG measured was RBD specific (*r* = 0.7198 [Fig. 3b]). This indicates, as expected, that antibodies against the RBD are involved in SARS-CoV-2 neutralization. Similarly, RBD-specific IgA and IgM correlated with neutralization (Fig. 3c and d), even though their abundance is highly diverse in the patient cohort (Table S1). In contrast, IgGs against the SARS-CoV-2 nucleocapsid measured by the Roche ECLIA poorly correlated with serum neutralization (*r* = 0.3249 [Fig. 3d]).

In conclusion, ELISAs or antibody tests, quantifying antibodies against the S protein and in particular the S-RBD, correlate best with patient serum neutralization.

### Antibodies against seasonal coronavirus 229E correlate with serum neutralization of SARS-CoV-2.

**FIG 4:**
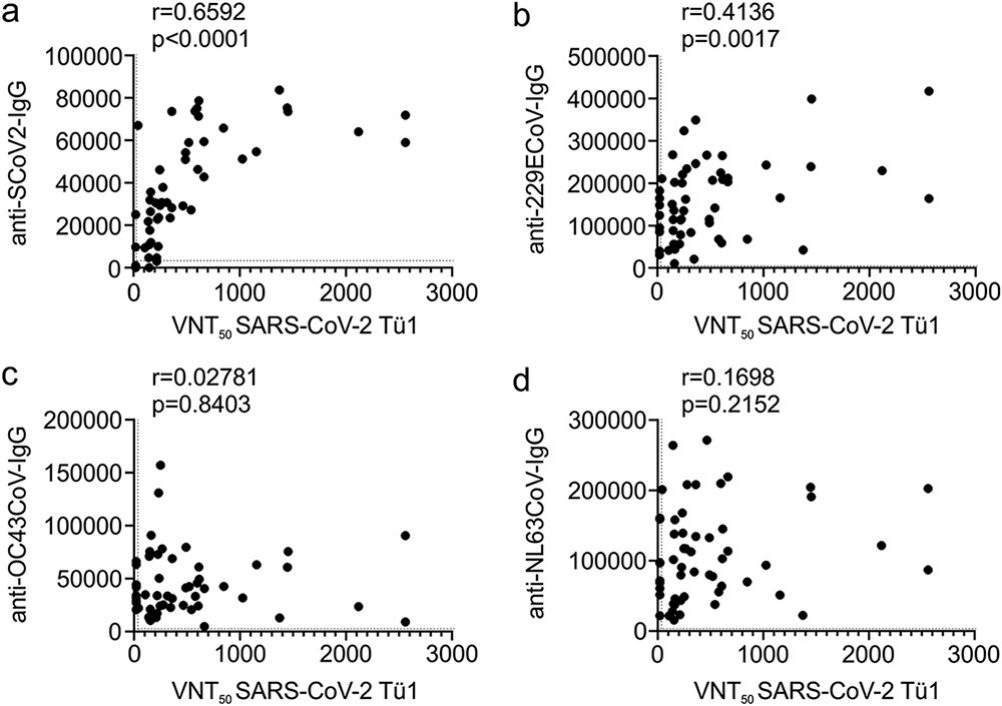
Correlation of antibodies against seasonal coronaviruses with serum VNT_50_. The VNT_50_ of each patient serum was correlated with the relative quantitative value of a throughput diagnostic Western blot detection system measuring CoV-specific IgG against SARS-CoV-2 (a), CoV-229E (b), CoV-OC43 (c), or CoV-NL63 (d). Dotted lines indicate the respective assay thresholds defined as positive. Correlations are calculated as Pearson’s *r*.

It is a matter of ongoing debate if antibodies against seasonal coronaviruses might confer cross-protection against SARS-CoV-2. To gain insight into this question, we employed a quantitative and high-throughput Western blot-based detection system identifying the bulk of IgGs against a specific coronavirus (16). As expected and in line with our previous data (Fig. 3a and b), IgG against SARS-CoV-2 correlated with VNT_50_ (*r* = 0.6592 [Fig. 4a]). Of note, IgG against the seasonal coronavirus 229E was modestly associated with VNT_50_ (*r* = 0.4136, *P* = 0.0017) (Fig. 4b), indicating that this class of antibodies might support SARS-CoV-2 neutralization. Remarkably, this effect was specific for 229E and not observed for seasonal coronavirus OC43 (Fig. 4c) or NL63 (Fig. 4d).

## DISCUSSION

Recent studies assessed the development of virus-specific antibodies in various cohorts of COVID-19 convalescent individuals (12, 13, 19–21). Overall, the published data are in accordance with ours, showing that the vast majority of individuals develop SARS-CoV-2-specific antibodies. Furthermore, SARS-CoV-2-specific IgGs were more prevalent than IgMs (20, 21), a finding that we confirm and extend to the abundance of SARS-CoV-2-specific IgA. Using pseudovirus-based neutralization assays, Robbiani et al. (20) also report that there is a correlation between VNT_50_ and antibodies against the S-RBD. Furthermore, males had significantly higher neutralizing activity than females, a finding which is also supported by our data (Fig. 2c). Even though, with 49 patients, our cohort size is limited, we extend the abovementioned studies in several aspects. First, for all our neutralization experiments we employed a fully infectious clinical SARS-CoV-2-isolate on a human cell line. Second, we performed a comprehensive comparison of several serological tests to delineate correlates of SARS-CoV-2 neutralization. This revealed that NC-specific antibodies poorly correlate with serum virus neutralization. In contrast, as supported by the findings of Robbiani et al. (20) and Ju et al. (22), RBD-specific IgGs correlate best with serum neutralization (Fig. 3b). In this context, it is noteworthy that S-RBD-specific IgA and IgM also showed a moderately high degree of correlation with the VNT_50_ (Fig. 3c and d), indicating that these antibodies, even though their abundance was highly diverse in our patient cohort, might contribute to serum neutralization.

A phenomenon that is critically discussed is the potential enhancement of infection by nonneutralizing antibodies (ADE) (14). For our VNT assays, we are using human cells expressing a diverse set of F_c_ receptors and directly assess the rate of infected cells by immunofluorescence or reporter gene expression. Hence, we should be able to detect enhancement of infection by serum that is not or only poorly neutralizing and in higher dilution ranges. However, in none of our 49 sera we observed ADE at any of the dilutions tested, indicating that antibodies generated in the natural context of SARS-CoV-2 infection are unlikely to contribute to severity of infection. This is in line with the absence of a correlation between the number of symptoms and VNT_50_ in our patients (Fig. 2d). On the other hand, our cohort is biased due to the fact that none of the patients was hospitalized. Therefore, it will be important to analyze if ADE plays a potential role in severe cases of COVID-19.

Until now, it was unclear if antibodies against seasonal coronaviruses that are highly prevalent within the human population play a role in SARS-CoV-2 neutralization. We employed an innovative throughput Western blot system to concomitantly detect antibodies specific against SARS-CoV-2 as well as the seasonal coronaviruses 229E, OC43, and NL63 (16). In fact, 100% of individuals included in our study had antibodies against the three seasonal coronaviruses with high diversity in relative numbers (see Table S1 in the supplemental material). Correlating the latter with our VNT_50_ values revealed a significant association of 229E-specific IgGs with the ability of patient sera to neutralize SARS-CoV-2 infection (Fig. 4b). This effect was 229E specific since none of the other seasonal coronaviruses showed such an association (Fig. 4c and d). While it is clear that 229E-specific IgGs are not sufficient to confer cross-protection against SARS-CoV-2, our data imply, based on correlation analyses, that the prevalence of such antibodies might assist in the neutralization of SARS-CoV-2. This hypothesis is in line with the observation that antibodies directed against the RBD of SARS or Middle East respiratory syndrome (MERS) virus alone are not sufficient to inactive SARS-CoV-2 (22). It will be highly interesting to analyze if the epidemiology of seasonal coronaviruses is a determinant of COVID-19 severity, with the implication that in areas with a high prevalence of antibodies against 229E, mortality is decreased.

In summary, we provide evidence for several correlates of SARS-CoV-2 neutralization by patient serum using a relevant virus-neutralization test. Even though S-RBD-specific IgGs correlate best with serum neutralization, it is clear that multiple factors contribute to a potent neutralizing antibody response. This might include subclasses of S-specific antibodies as, for instance, IgM and IgA as well as the antibody response elicited against the seasonal coronavirus 229E. This makes it particularly difficult to define singular serological correlates of immune protection as discussed in the context of COVID-19 “immunity passports.” Furthermore, such an approach neglects other potentially essential factors of immune protection such as T-cell-mediated immunity (23, 24) and the innate immune response (25).

## MATERIALS AND METHODS

### Study participants and sample processing.

Blood was drawn from potential blood donors for reconvalescent plasma therapy after written consent at the Clinical Transfusion Medicine, Tübingen, Germany, between 4 April and 12 May 2020, under the guidelines of the local ethics committees, 222/2020BO. The study cohort consisted of 49 patients older than 18 years, who either provided a PCR-confirmed diagnosis of SARS-CoV-2 (*n* = 46) or were symptomatic and close contacts of positively diagnosed COVID-19 patients (partners tested positive) (*n* = 3). All patients were nonhospitalized with asymptomatic to mild courses of disease, and they were fully convalescent, showing no symptoms on the day of blood donation. Basic demographic information was collected including age and sex, as well as self-perceived symptoms (cough, fever, limb pain and headache, diarrhea, and loss of taste). In addition, blood from four healthy donors and one hospitalized patient was collected (see Table S1 in the supplemental material). Serum samples were stored at −80°C.

### Cell culture.

Caco-2 (human colorectal adenocarcinoma) cells were cultured at 37°C with 5% CO_2_ in Dulbecco modified Eagle medium (DMEM) containing 10% fetal calf serum (FCS), with 2 mM l-glutamine, 100 μg/ml penicillin-streptomycin, and 1% nonessential amino acids (NEAA).

### Viruses.

A throat swab sample collected in March 2020 at the diagnostic department of the Institute for Medical Virology and Epidemiology of Viral Diseases, University Hospital Tübingen, from a SARS-CoV-2-positive patient was used to isolate the virus (200325_Tü1). Fifty microliters of patient material was diluted in medium, sterile filtrated, and used directly to inoculate 200,000 Caco-2 cells in a 6-well plate. At 48 hpi (hours postinfection) the supernatant was collected, centrifuged, and stored at −80°C. Supernatant as well as cell lysates from infected cells was tested by Western blotting using a SARS-CoV-2 anti-nucleocapsid protein (NP) specific antibody (GeneTex). The identity of the virus was confirmed by qRT-PCR via S and E gene amplification (RealStar SARS-CoV-2 RT-PCR; Altona, Germany) with a high viral load (cycle threshold [*C_T_*] values <17). An aliquot of the isolate was used to amplify the virus in a medium flask of Caco-2 cells (2 × 10^6^ cells) in 13 ml DMEM plus supplements and 5% FCS. At 48 hpi, the supernatant was centrifuged and stored in aliquots at −80°C.

The recombinant SARS-CoV-2 expressing mNeonGreen (icSARS-CoV-2-mNG) (26) was obtained from the World Reference Center for Emerging Viruses and Arboviruses (WRCEVA) at the UTMB (University of Texas Medical Branch). To generate icSARS-CoV-2-mNG stocks, Caco-2 cells were infected as described above, and the supernatant was harvested 48 hpi, centrifuged, and stored at −80°C.

Next-generation sequencing (NGS) was used to fully characterize isolates 200325_Tü1 and icSARS-CoV-2-mNG. Briefly, RNA was isolated from viral stocks by using the Qiagen DSP virus pathogen minikit on a QIAsymphony instrument (Qiagen, Hilden, Germany). The sequencing was performed at the NGS Competence Center Tübingen, UKT, Germany, and the data analysis was carried out at the Institute of Medical Genetics and Applied Genomics, University of Tübingen. The clinical isolate 200325_Tü1 was identified as belonging to the B.1.126 SARS-CoV-2 lineage while icSARS-CoV-2-mNG belongs to the A lineage, as expected.

For multiplicity of infection (MOI) determination, a titration using serial dilutions of both virus stocks (200325_Tü1 and mNG) was conducted. The number of infectious virus particles per milliliter was calculated as (MOI × cell number)/(infection volume), where MOI = −ln(1 − infection rate). To reach an infection rate of ∼20%, an MOI of 0.3 was used for SARS-CoV-2-200325_Tü1 and 1.1 for SARS-CoV-2-mNG.

### Enzyme-linked immunosorbent assays (ELISAs).

**(i) Euroimmun.** The Euroimmun SARS-CoV-2 ELISA (IgG) (Euroimmun, Lübeck, Germany) with the recombinant S1 target antigen of SARS-CoV-2 was performed according to manufacturer’s instructions in serum. Patient samples are diluted 1:101 in sample buffer. The included controls and calibrator in the test kit were used with each run. Results are given as ratios (optical density [OD] of control or clinical sample/OD of calibrator). According to the manufacturer, ratios were classified as negative (<0.8), borderline (≥0.8 to <1.1), and positive (≥1.1).

**(ii) Mediagnost.** IgG antibody detection directed to the S1-RBD SARS-CoV-2 in human sera using the Mediagnost test system was performed according to the manufacturer’s instructions. Briefly, these tests are two-step enzyme-linked immunosorbent assays. The solid phase consists of a 96-well microtiter plate (Greiner, Bio-One, Frickenhausen, Germany) that is coated with the recombinant SARS-CoV-2 spike protein S1. The antibodies from patients that are directed against SARS-CoV-2 S1 protein bind to the solid-phase-coated S1 protein. Next, a horseradish peroxidase (HRP)-conjugated goat anti-human IgG binds to the human IgG antibodies. The following step involves the substrate for the HRP being added, by which the substrate is converted from colorless into blue, and after addition of a stop solution, the color changes to yellow. The extinction of the yellow solution can be measured at a wavelength of 450 nm with reference at 620 nm. Increasing extinctions represent increasing amounts of antibodies to SARS-CoV-2 S1 protein. Samples showing extinctions that were three times higher than the negative control can be interpreted as being positive for anti-SARS-CoV-2 S1. IgA and IgM antibody detection directed to the SARS-CoV-2 S1 protein was made in analogy to the above-described IgG detection system except that the HRP-labeled detection antibody was directed against human IgA or human IgM antibodies. According to the manufacturer, negative, borderline, and positive ratios were classified as follows: <0.42, ≥0.42 to 0.7, and ≥0.7 for IgG, <0.33, ≥0.33 to 0.7, and ≥0.7 for IgA, and <0.87, ≥0.87 to 1.47, and ≥1.47 for IgM, respectively.

### Elecsys anti-SARS-CoV-2 (Roche).

For qualitative detection of anti-SARS-CoV-2 (IgG + IgM) antibodies, the electrochemiluminescence immunoassay (ECLIA) was performed using the fully automated Cobas E 6000/601 immunoassay analyzer (Roche Diagnostics, Mannheim, Germany). This assay targets recombinant SARS-CoV-2 nucleocapsid (NC) protein. Two calibrators are used (Cal1 nonreactive, cutoff index [COI] of 0.101; and Cal2 reactive, COI of 1.2) in the double antigen-sandwich-based assay (SARS-CoV-2 recNC biotin label, and SARS-CoV-2 recNC ruthenium complex label). Twenty microliters each of sera and reference solutions were used, and immune complexes were fixed to streptavidin-coated microparticles. Readout is given in relative light units in the form of cutoff index (COI, signal/cutoff). The Elecsys reagents derived from lot 49500101. For negative control (<150% Cal1), we used pooled sera from 100 mothers at birth of the 2012 Tuebingen congenital cytomegalovirus (CMV) study. Furthermore, we used a negative-control serum from a direct COVID-19 contact person, repeatedly negatively tested for SARS-CoV-2 RNA and nucleocapsid-specific antibodies without any symptoms during and after a 1-week close exposure. For positive control, we used a dilution series of a serum from a reconvalescent student infected symptomatically (fever, cough, loss of smell) who tested positive for viral RNA and NC-specific antibodies. The COIs ranged from 100 to 1. If the numeric COI result was ≥1.0, the serum was diagnosed as reactive, and COIs of <1.0 were attributed as nonreactive. COI values of the positive controls were stable over at least 2 months.

### Multiplexed detection of anticoronavirus antibodies.

Whole-viral-protein lysates from 229E, OC43, and NL63 (ZeptoMetrix Corp.) and from SARS-CoV-2 were used for DigiWest as described previously (16). Viral protein lysates were used for denaturing gel electrophoresis and Western blotting using the NuPAGE system. Blot membranes were washed with PBST (0.1% Tween 20, phosphate-buffered saline [PBS]), and membrane-bound proteins were biotinylated by adding 50 μM *N*-hydroxysuccinimide (NHS)–PEG12–biotin (Thermo Fisher Scientific) in PBST for 1 h. After washing in PBST, membranes were dried overnight. Subsequently, the Western blot lanes were cut into 96 strips of 0.5-mm width and were transferred to a 96-well plate (Greiner Bio-One). For protein elution, 10 μl of elution buffer was added to each well (8 M urea, 1% Triton X-100 in 100 mM Tris-HCl, pH 9.5). The protein eluates were diluted with 90 μl dilution buffer (5% bovine serum albumin [BSA] in PBST, 0.02% sodium azide). Neutravidin-coated MagPlex beads (Luminex) of a distinct color identity (ID) were added to the protein eluates, and binding was allowed overnight; 500 μM PEG12-biotin in PBST was added to block remaining neutravidin binding sites. The bead-containing fractions were pooled, and thereby the original Western blot lanes were reconstituted. Beads were washed in PBST and resuspended in storage buffer (1% BSA, 0.05% azide, PBS). The generated bead set represents the proteomes of the four coronaviruses (SARS-CoV-2, OC43, 229E, NL63), and reactivity against all proteins can be tested in one assay.

For serum incubation, 5 μl of the bead mix was equilibrated in 50 μl serum assay buffer (blocking reagent for ELISA [Roche] supplemented with 0.2% milk powder, 0.05% Tween 20, and 0.02% sodium azide, 25% Low Cross buffer [Candor Bioscience], 25% IgM-reducing agent buffer [immunochemistry]). Serum assay buffer was discarded, and 30 μl of diluted patient serum (1:200 in serum assay buffer) was added and incubated for 2 h at room temperature (RT) on a shaker. After washing in PBST, 30 μl of phycoerythrin-labeled anti-human IgG secondary antibody (diluted 1:200 in serum assay buffer; Dianova) was added and incubated for 45 min at 23°C. The beads were washed twice with PBST, and readout was performed on a Luminex FlexMAP 3D.

The DigiWest analysis tool was used to assess serum reactivity against the viral proteins (16). Virus protein-specific peaks were identified, and average fluorescence intensity (AFI) values were calculated by integration of peak areas.

### Neutralization assay.

For neutralization experiments, 1 × 10^4^ Caco-2 cells/well were seeded in 96-well plates the day before infection in medium containing 5% FCS. Cells were coincubated with SARS-CoV-2 clinical isolate 200325_Tü1 at an MOI of 0.3 and patient sera in serial 2-fold dilutions from 1:20 up to 1:2,560. At 48 hpi cells were fixed with 80% acetone for 5 min, washed with PBS, and blocked for 30 min at room temperature (RT) with 10% normal goat serum (NGS). Cells were incubated for 1 h at RT with 100 μl of serum from a hospitalized convalescent donor in a 1:1,000 dilution and washed 3 times with PBS. One hundred microliters of goat anti-human Alexa 594 (1:2,000) in PBS was used as secondary antibody for 1 h at RT. Cells were washed 3 times with PBS and counterstained with 1:20,000 DAPI solution (2 mg/ml) for 10 min at RT. For quantification of infection rates, images were taken with the Cytation3 (BioTek) and DAPI-positive and Alexa 594-positive cells were automatically counted by the Gen5 software (BioTek).

Alternatively, Caco-2 cells were coincubated with the SARS-CoV-2 strain icSARS-CoV-2-mNG at an MOI of 1.1 and patient sera in serial 2-fold dilutions from 1:40 up to 1:5,120. At 48 hpi cells were fixed with 2% PFA and stained with Hoechst 33342 (1-μg/ml final concentration) for 10 min at 37°C. The staining solution was removed and exchanged for PBS. For quantification of infection rates, images were taken with the Cytation3 (BioTek) and Hoechst-positive and mNG-positive cells were automatically counted by the Gen5 software (BioTek). Virus-neutralizing titers (VNT_50_s) were calculated as the half-maximal inhibitory dose (ID_50_) using 4-parameter nonlinear regression (GraphPad Prism).

### Software and statistical analysis.

GraphPad Prism 8.0 was used for statistical and correlation analyses and to generate graphs. Figures were generated with CorelDrawX7. Other software used included Gen5 v.3.04.
